# Ultrasound Responsive Nanovaccine Armed with Engineered Cancer Cell Membrane and RNA to Prevent Foreseeable Metastasis

**DOI:** 10.1002/advs.202301107

**Published:** 2023-04-25

**Authors:** Wenqi Sun, Panpan Ji, Tian Zhou, Zhelong Li, Changyang Xing, Liang Zhang, Mengying Wei, Guodong Yang, Lijun Yuan

**Affiliations:** ^1^ Department of Ultrasound Diagnostics Tangdu Hospital Fourth Military Medical University Shaanxi 710038 China; ^2^ The State Laboratory of Cancer Biology Department of Biochemistry and Molecular Biology Fourth Military Medical University Shaanxi 710032 China; ^3^ Department of Digestive Surgery Xijing Hospital Fourth Military Medical University Shaanxi 710032 China

**Keywords:** alternative splicing, antigen presentation, cancer vaccine, sonosensitizer

## Abstract

Cancer vaccine has been considered as a promising immunotherapy by inducing specific anti‐tumor immune response. Rational vaccination at suitable time to efficiently present tumor associated antigen will boost tumor immunity and is badly needed. Here, a poly (lactic‐*co*‐glycolic acid) (PLGA)‐based cancer vaccine of nanoscale is designed, in which engineered tumor cell membrane proteins, mRNAs, and sonosensitizer chlorin e6 (Ce6) are encapsulated at high efficiency. The nanosized vaccine can be efficiently delivered into antigen presentation cells (APCs) in lymph nodes after subcutaneous injection. In the APCs, the encapsulated cell membrane and RNA from engineered cells, which have disturbed splicing resembling the metastatic cells, provide neoantigens of metastatic cancer in advance. Moreover, the sonosensitizer Ce6 together with ultrasound irradiation promotes mRNA escape from endosome, and augments antigen presentation. Through 4T1 syngeneic mouse model, it has been proved that the proposed nanovaccine is efficient to elicit antitumor immunity and thus prevent cancer metastasis.

## Graphical abstract

The designed nanosized cancer vaccine, in which cell membrane proteins and RNAs from engineered cells with splicing disturbed, and sonosensitizer Ce6 are encapsulated into the PLGA‐based nanoparticles, could be efficiently delivered into lymph nodes. Assisted by ultrasound irradiation, the vaccine elicits strong immune response to inhibit tumor growth and prevent metastasis.

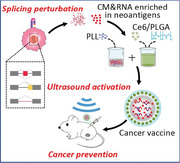

## 1 Introduction

Cancer vaccine has been developed as a novel promising cancer treatment for many years.^[^ ^1^ ^]^ Currently, preventative and therapeutic vaccines are the two kinds of cancer vaccines available. Preventive vaccines usually protect the recipients from the infection of oncoviruses, while therapeutic vaccines elicit immune response attacking cancer cells.^[^ ^2^, ^3^ ^]^ Due to the reasons including lack of effective vaccine and disabled immune system in cancer, the full potential of the strategy has yet to be realized.^[^ ^4^ ^]^ Hence, novel strategies to booster the effectiveness of cancer vaccine are badly needed.

Rational design of effective cancer vaccines to induce lasting antitumor immune response and avoid adverse reactions depends on understanding of tumor‐associated antigens, the immune response during tumor progression and development of novel antigen delivery technologies.^[^ ^5^ ^]^ The most commonly studied class of tumor‐specific antigens (TSAs) are those derived from non‐synonymous single‐nucleotide variants (SNVs), or SNV neoantigens. Besides, non‐SNV genomic sources, have recently been evaluated, including antigens derived from mutational frameshifts, splice variants, and other processes. Notably, some of these alternative TSAs may being shared by multiple tumors, allowing for universal, off‐the‐shelf therapies.^[^ ^6^ ^]^ Alternative splicing (AS) is a process by which pre‐mRNAs are spliced into mature mRNAs by spliceosome and splicing factors. The rearrangement of exons and introns by AS contributes to protein diversity and regulates biological activities. Recently, some mutations in spliceosome or splicing factors have been proved in many solid cancers including breast cancer, melanoma, and lung cancer, and is believed to disturb normal mRNA splicing. During the process of tumor recurrence and metastasis, alternative splicing plays a crucial role.^[^ ^7^ ^]^ For example, SF3B1 is a subunit of the spliceosome factor 3b complex, and its mutations promote malignancy and result in poor prognosis.^[^ ^8^ ^]^ SRSF1, which is a serine/arginine‐rich protein, is another oncogenic splicing factor, and overexpressed in breast cancer. It has been confirmed that SRSF1 expression is positively associated with breast cancer grade and acts as an indicator of poor prognosis. Besides, SNRPA1 has been confirmed as a prometastatic splicing enhancer in breast cancer, and splicing regulator DDX17 promotes extrahepatic metastasis of hepatocellular carcinoma.^[^ ^9^, ^10^ ^]^ With the advancement of sequencing technology, there are more alternative splicing events been discovered in cancer. The aberrant alternative splicing not only produces oncogenic proteins but also neoantigens. For example, splicing factor SF3B1 has been shown to generate immunogenic neoantigens in uveal melanoma.^[^ ^11^ ^]^ Accordingly, animal researches have revealed that modulation of RNA splicing by chemical compounds can generate neoantigens, which in turn prime CD8^+^ T cells and enhance the anti‐tumor immune response.^[^ ^12^ ^]^ During cancer progression, alternative splicing is found to be disturbed and accumulation of neoantigens are expected to elicit anti‐tumor immunity. However, the immune system is also destroyed during tumor progression. Thus, earlier exposure of the antigens generated by aberrant alternative splicing might be a promising method to prevent cancer recurrence and metastasis.

Dendritic cells (DCs) are professional antigen‐presenting cells and play a central role in the initial activation of antitumor immunity. The uptake and presentation of antigens by DCs is one of the most crucial events in the induction of adaptive immune response.^[^ ^13^ ^]^ To elicit an effective vaccination, cancer antigens delivered by vaccine have to be taken up by DCs and presented by major histocompatibility complex (MHC) I for CD8^+^ T cells priming. For the protein antigen composited vaccine, the immature DCs take up the vaccine and undergoes a maturation process. Then, they migrate to the draining lymph node and simultaneously cross‐present the antigens for priming of naive CD8^+^ T cells.^[^ ^14^, ^15^ ^]^ The cross‐presentation is tightly regulated and disabled with tumor progression.^[^ ^16^ ^]^ In contrast, mRNA vaccines, which can simulate natural MHC I type presentation, have natural superiority when applicated in cancer therapy. However, the fate of the mRNA after endosome‐engulfing and escape from the endosomal–lysosomal pathway is prerequisite for the following RNA translation and thus antigen presentation.^[^ ^17^ ^]^ Currently, it has been found that sonodynamic therapy could be used to facilitate the endosome/lysosome escape.^[^ ^18^, ^19^ ^]^

**Scheme 1:**
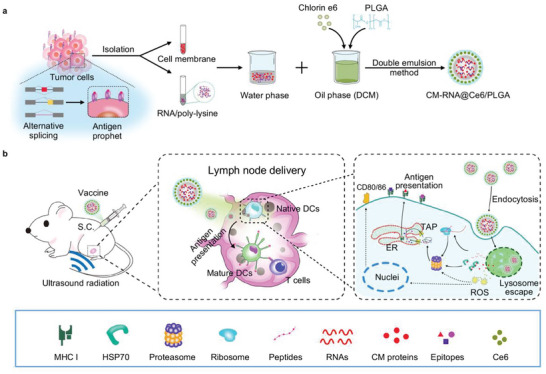
a) Schematic illustration showing the preparation steps of cancer vaccine. b) Schematic of vaccine delivery in lymph node and intercellular antigen presentation.

Generally, in order to design an effective cancer vaccine, tumor specific antigens, healthy immune system, and appropriate timing of immunization should be considered. In the present study, we have designed a poly (lactic‐*co*‐glycolic acid) (PLGA)‐based cancer vaccine of nanoscale, in which cell membrane proteins, mRNAs from engineered tumor cells with disturbed alternative splicing, and sonosensitizer chlorin e6 (Ce6) are encapsulated at high efficiency. The nanosized vaccine can be efficiently delivered into antigen presentation cells (APCs) in lymph nodes after subcutaneous injection. In the APCs, the encapsulated cell membrane and RNA from engineered cells, which have disturbed splicing resembling the metastatic cells, provide neoantigens of metastatic cancer in advance. Moreover, the sonosensitizer Ce6 together with ultrasound irradiation promotes mRNA escape from endosome, and augments antigen presentation. Through 4T1 syngeneic mouse model, we have proved that the proposed nanovaccine is efficient to elicit antitumor immunity and thus prevent cancer metastasis (**Scheme** **1**).

## 2 Results and Discussion

### 2.1 Preparation and Characterization of CM‐RNA@Ce6/PLGA

**Figure 1:**
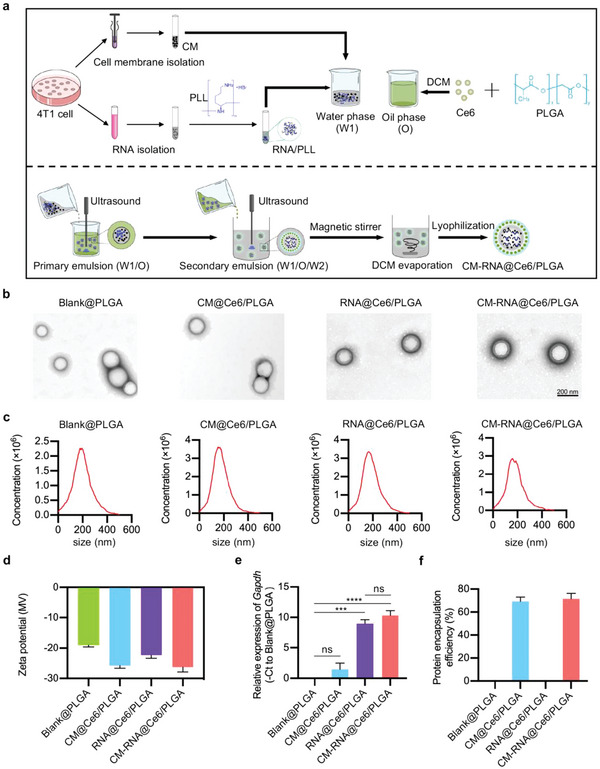
Preparation and characterization of fabricated nanoparticles. a) Schematic illustration showing the procedure of cell membrane and RNA and cancer vaccine preparation with double emulsion method. b) TEM image of Blank@PLGA, CM@Ce6/PLGA, RNA@Ce6/PLGA, and CM‐RNA@Ce6/PLGA. Scale bar = 200 nm. c) Size distribution of Blank@PLGA, CM@Ce6/PLGA, RNA@Ce6/PLGA, and CM‐RNA@Ce6/PLGA. d) Zeta potential of Blank@PLGA, CM@Ce6/PLGA, RNA@Ce6/PLGA, and CM‐RNA@Ce6/PLGA. *n* = 3. e) qPCR analysis of *Gapdh* expression in lysates of Blank@PLGA, CM@Ce6/PLGA, RNA@Ce6/PLGA, and CM‐RNA@Ce6/PLGA. *n* = 3. f) The protein encapsulation efficiency of Blank@PLGA, CM@Ce6/PLGA, RNA@Ce6/PLGA, and CM‐RNA@Ce6/PLGA measured by BCA assay. *n* = 3. Data are represented by mean ± SEM of three replicates. Statistical significance was determined by one‐way ANOVA with Tukey's post hoc test. ***p < 0.001, ****p < 0.0001. ns, not significant.

Cancer cell membrane and RNA contain the full array of neoantigens.^[^ ^20^ ^]^ In this study, we aimed to design a nanovaccine armed with cancer cell membrane and RNA, and sonosensitizer, namely CM‐RNA@Ce6/PLGA. The preparation process of CM‐RNA@Ce6/PLGA is illustrated in **Figure** **1**a. As a proof‐of‐concept, 4T1 cells were selected. In brief, cell membrane and RNAs from 4T1 cells were isolated respectively. Coomassie blue staining of the gel and Western blotting analysis of membrane biomarkers Pan Cadherin and Na^+^/K^+^ ATPase, suggested the cell membrane was successfully isolated (Figure S1, Supporting Information). Isolated RNAs were additionally complexed by incubation with poly‐l‐lysine (PLL) to improve loading efficiency and to avoid damage during vaccine preparation, as nucleic acids could form a larger aggregate in solution with PLL.^[^ ^21^ ^]^ As shown in Figure S2 (Supporting Information), when PLL/RNA ratio reached 2.5, RNAs were efficiently complexed with PLL, as seen by the retardation of the RNA in the gel. Then, CM‐RNA@Ce6/PLGA nanoparticles were prepared via double emulsion method. The transmission electron microscopy (TEM) images showed highly dispersed nanoparticles with spherical shape. Notably, there were no obvious change in shape and size after cell membrane coating, RNA loading, or Ce6 encapsulation (Figure 1b). Nanoparticle tracking analysis (NTA) further revealed that the nanoparticles had an average diameter of about 200 nm (Figure 1c). The surface zeta potential of the nanoparticles was also measured, with −18.97 mV for Blank@PLGA, −25.82 mV for CM@Ce6/PLGA, −22.33 mV for RNA@Ce6/PLGA, and −26.32 mV for CM‐RNA@Ce6/PLGA respectively (Figure 1d). The differences of surface potential may be caused by negative charged cell membrane and loaded Ce6. Absolute qPCR analysis of *Gapdh* and BCA assay of protein concentration revealed that both RNA and cell membrane were successfully loaded into the nanoparticles (Figure 1e,f). UV–Vis spectrophotometer analysis of Ce6 absorbance also revealed that Ce6 could be loaded on the nanoparticles (Figure S3, Supporting Information). The encapsulation efficiency of RNA and Ce6 in RNA@Ce6/PLGA were 31.5% and 15.2% respectively, when 600 µg RNA in 300 µL water phase and 4 mg Ce6 in 1 mL oil phase solution mixed together.

### 2.2 Delivery of CM‐RNA@Ce6/PLGA into Antigen Presentation Cells

**Figure 2:**
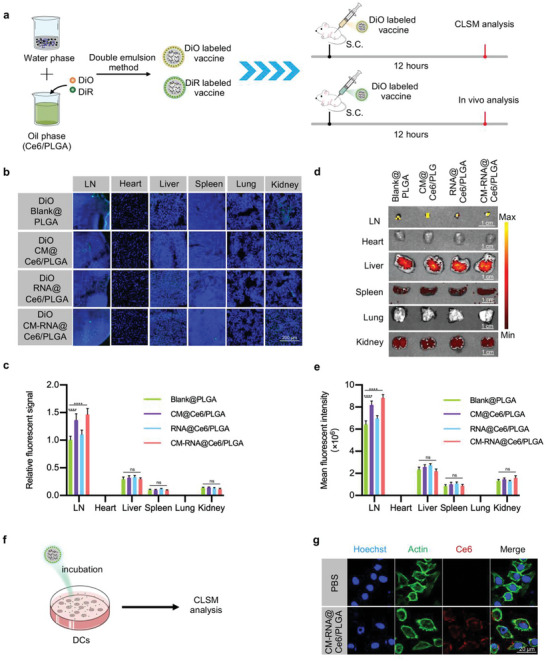
In vitro and in vivo delivery of the fabricated nanoparticles. a) Schematic showing the preparation of DiO and DiR labeled nanoparticles and animal experiment procedures. b) Representative confocal microscope images of the distribution of DiO labeled nanoparticles in the lymph nodes and other main organs 12 h after subcutaneous injection. *n* = 3. Scale bar = 200 µm. c) Fluorescent statistics of (b). d) Representative IVIS analysis of the distribution of DiR labeled nanoparticles in lymph nodes and other main organs 12 h after subcutaneous injection. *n* = 3. Scale bar = 1 cm. e) Fluorescent statistics of (d). f) Schematic showing the cell uptake experiment. g) Confocal fluorescence images showing the uptake of nanoparticles by DCs. Cell nuclei were stained with Hoechst 33342 (blue), and cytoskeletons were stained with Actin‐Tracker Green (green). Scale bar = 20 µm. Data shown are representative images of three different experiments. Statistical significance was determined by two‐way ANOVA with Tukey's post hoc test. ****p < 0.0001. ns, not significant.

We next explored whether CM‐RNA@Ce6/PLGA and control nanoparticles could be efficiently delivered into APCs, by investigating the in vivo distribution of CM‐RNA@Ce6/PLGA after subcutaneous injection (s.c.). The nanoparticles were labeled with 3,3′‐dioctadecyloxacarbocyanine perchlorate (DiO) or 1,1′‐dioctadecyl‐3,3,3′,3′‐tetramethylindotricarbocyanine iodide (DiR) during preparation for confocal laser scanning microscopy (CLSM) analysis and in vivo animal florescent imaging respectively (**Figure** **2**a). CLSM analysis showed high delivery efficiency of Blank@PLGA, CM@Ce6/PLGA, RNA@Ce6/PLGA, and CM‐RNA@Ce6/PLGA into lymph nodes (Figure 2b,c). Strikingly, there were significantly more nanoparticles enriched in lymph nodes for the CM@Ce6/PLGA and CM‐RNA@Ce6/PLGA, which should be explained by the lymph node homing effects of the cancer cell membrane.^[^ ^20^ ^]^ Notably, there were also slight fluorescent signals detected in the liver, spleen, and kidney, but not in heart and lung (Figure 2b,c). The in vivo fluorescent images had the same results (Figure 2d,e). We next incubated CM‐RNA@Ce6/PLGA with DC2.4 cells for 12 h and examined the endocytosis by CLSM (Figure 2f). As expected, CM‐RNA@Ce6/PLGA was found efficiently engulfed by DC2.4 cells, as seen by the abundant Ce6 signal in the cells (Figure 2g). These results confirmed the lymph node enrichment ability of CM‐RNA@Ce6/PLGA, and a small amount of vaccine in other organs helped to increase the safety.

### 2.3 CM‐RNA@Ce6/PLGA Boosting Antigen Presentation under Ultrasound Radiation

**Figure 3:**
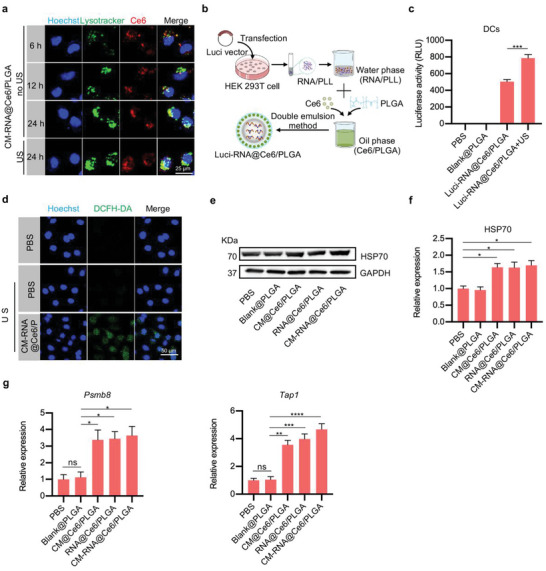
Ultrasound irradiation promotes endosome/lysosome escape and enhances antigen presentation associated factors. a) Confocal microscope images of colocalization of CM‐RNA@Ce6/PLGA and DCs. The lysosomes were stained with Lyso‐Tracker Green, and nuclei were stained with Hoechst 33342. Scale bar = 25 µm. b) Schematic of Luci‐RNA@Ce6/PLGA preparation. c) Luciferase activity in cells treated as indicated. *n* = 3. d) Confocal microscope images of ROS generated in DCs. ROS was stained with DCFH‐DA, and nuclei were stained with Hoechst 33342. Scale bar = 50 µm. e) Western blot analysis of HSP70 expression in DCs treated as indicated. GAPDH served as internal control. f) Statistics of Western blot data. *n* = 3. g) mRNA expression tested by qPCR. *n* = 3. Data are represented by mean ± SEM. Statistical significance was determined by one‐way ANOVA with Tukey's post hoc test. *p < 0.05, **p < 0.01, ***p < 0.001, ****p < 0.0001. ns, not significant.

Lysosomes are critical organelles for exogenous antigen processing and presentation,^[^ ^22^ ^]^ while endosome/lysosome escape is essential for RNA translation.^[^ ^23^ ^]^ Next, we investigated whether CM‐RNA@Ce6/PLGA could simultaneously deliver protein cargos to lysosome for degradation and functional RNAs to cytosol for translation after being uptaken by DCs. In order to track the nanoparticles in DCs, DCs were first incubated with 0.5 mg mL^−1^ nanoparticles for 6 h and then switched to fresh medium. In the US group, cells were additionally treated with ultrasound (1 W cm^−2^, 1 min) at 12 h. As shown in **Figure** **3**a, the nanoparticles gradually accumulated in the lysosomes with time. However, ultrasound treatment induced significant nanoparticles escaped from the endosome/lysosome to cytoplasm. To confirm that RNAs loaded into CM‐RNA@Ce6/PLGA could be translated as proteins, we constructed Luci‐RNA@Ce6/PLGA, in which the RNA was derived from the luciferase mRNA overexpressing cells (Figure 3b). DCs were treated with Luci‐RNA@Ce6/PLGA and control nanoparticles and cultured for 48 h with or without ultrasound irradiation. The luciferase activity was then detected. Optimization experiments revealed that ultrasound irradiation at 1 W cm^−2^ with 2 µg mL^−1^ Ce6 loaded had no obvious cell toxicity (Figure S4, Supporting Information), and in the following experiments, the parameters were selected. The results showed that the nanoparticle could deliver functional luciferase mRNA for translation, while ultrasound irradiation further augmented the translation efficiency (Figure 3c), possibly via facilitating endosome/lysosome escape.^[^ ^24^ ^]^

**Figure 4:**
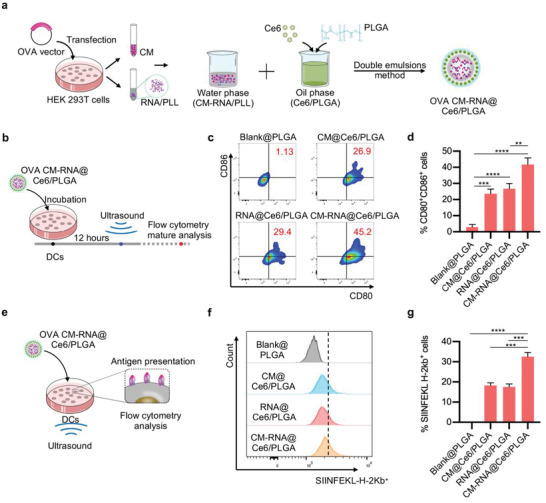
Ultrasound irradiation boosts DCs maturation and antigen presentation. a) Schematic depicting OVA CM‐RNA@Ce6/PLGA preparation. b) Schematic diagram showing the experimental procedure of DCs maturation analysis. c) Representative flow cytometry data showing the antigen presentation analysis. *n* = 3. d) Statistics of (c). e) Schematic diagram showing the antigen presentation analysis. f) Representative flow cytometry data of SIINFEKL peptide presented by DCs. *n* = 3. g) Statistics of (f). Data are represented by mean ± SEM. Statistical significance was determined by one‐way ANOVA with Tukey's post hoc test. **p < 0.01, ***p < 0.001, ****p < 0.0001.

Reactive oxygen species (ROS) and heat shock protein 70 (HSP70) have been confirmed as a stimulator for DCs maturation and regulate the expression of many genes related to antigen presentation.^[^ ^25^, ^26^, ^27^ ^]^ Hence, we explored whether our strategy could increase the level of ROS and HSP70 in DCs. The CLSM and western blot analysis indicated that CM‐RNA@Ce6/PLGA together with ultrasound treatment could stimulate ROS generation and HSP70 expression in DCs (Figure 3d–f). Meanwhile, *Psmb8* and *Tap1*, which are related to antigen presentation, were also increased (Figure 3g). Next, we chose ovalbumin (OVA) as model protein to explore the vaccination effects of CM‐RNA@Ce6/PLGA. Cell membrane and RNA were isolated from OVA overexpressing cells and loaded into the nanoparticles (**Figure** **4**a), namely OVA CM‐RNA@Ce6/PLGA. As expected, OVA CM‐RNA@Ce6/PLGA together with ultrasound showed highest ability to elicit cell maturation (Figure 4b–d), while ultrasound treatment alone did not have any effects (Figure S5, Supporting Information). Further, we evaluated the antigen presentation ability of OVA CM‐RNA@Ce6/PLGA (Figure 4e). The ratio of cells displaying SIINFEKL bound to H‐2Kb of MHC class I was significantly higher in OVA CM‐RNA@Ce6/PLGA in combination with ultrasound treatment (Figure 4f,g). All these results confirmed that CM‐RNA@Ce6/PLGA together with ultrasound irradiation could booster antigen presentation by DCs.

### 2.4 Anti‐tumor Effects of CM‐RNA@Ce6/PLGA In Vivo

**Figure 5:**
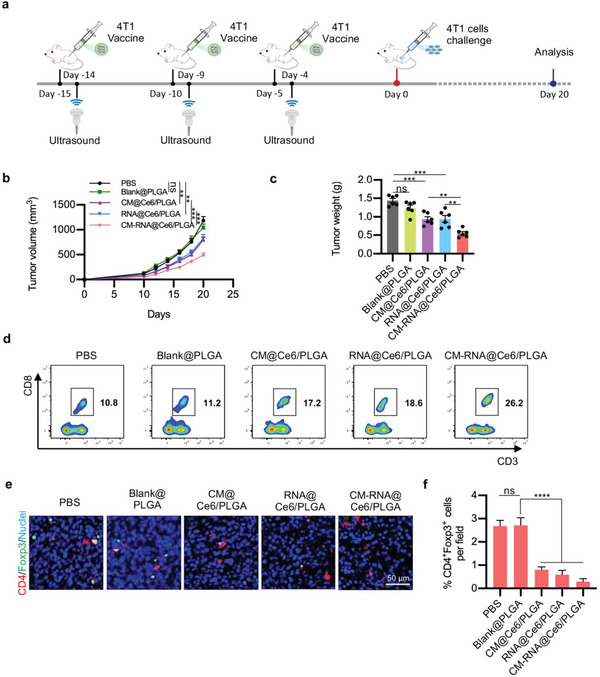
Inhibition of tumor growth by cancer vaccine. a) Schematic illustration showing the vaccination schedule and tumor challenge. b) Growth curves for 4T1 tumors on mice after immunity by different nanovaccines. *n* = 6. Statistical significance was determined by two‐way ANOVA with Tukey's post hoc test. c) The tumor weight in different groups. *n* = 6. d) Flow cytometry data showing the infiltrating CTLs at the tumor tissues with different treatments. e) Immunofluorescent staining of indicated markers in tumor tissues with different treatments. *n* = 6. f) Statistics of (e). Data are represented by mean ± SEM. Statistical significance was determined by one‐way ANOVA with Tukey's post hoc test. **p < 0.01, ***p < 0.001, ****p < 0.0001.

The antitumor efficacy of CM‐RNA@Ce6/PLGA was then evaluated. As illustrated in **Figure** **5**a, mice were immunized three times through s.c. with PBS, Blank@PLGA, CM@Ce6/PLGA, RNA@Ce6/PLGA, or CM‐RNA@Ce6/PLGA (2 mg in 100 µL PBS). After vaccination, 4T1 cells were inoculated in BALB/c mice. As expected, CM‐RNA@Ce6/PLGA had the maximum effect to inhibit tumor growth, while CM@Ce6/PLGA and RNA@Ce6/PLGA had slighter inhibition effects (Figure 5b,c). Consisted with tumor growth inhibition, compared with other groups, there were more infiltrating cytotoxic T lymphocytes (CTLs) in tumor tissues (Figure 5d) and less CD4^+^Foxp3^+^ Treg cells (Figure 5e) in the CM‐RNA@Ce6/PLGA treatment group. All these in vivo experiments confirmed that CM‐RNA@Ce6/PLGA significantly inhibited tumor growth via immune elicitation.

### 2.5 Function of SRSF1 in Metastasis and Neoantigen Generation

**Figure 6:**
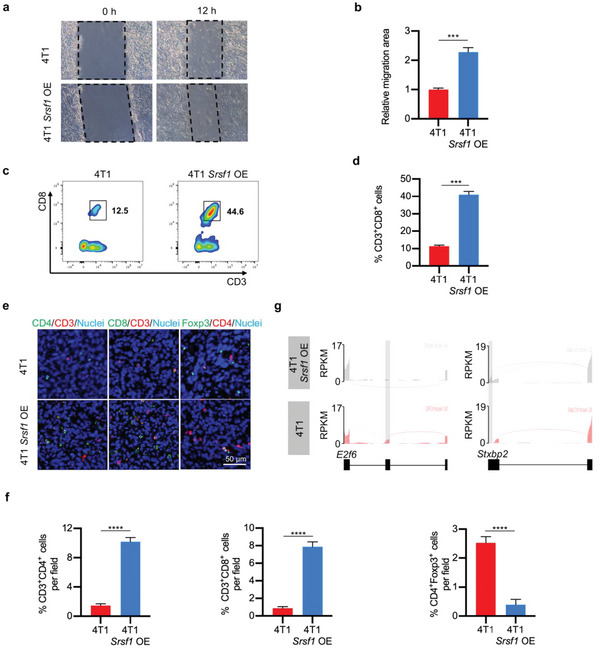
SRSF1 promotes cancer cell migration and enhances immunogenicity. a) Representative images of wound healing assay. *n* = 3. b) Statistics of (a). c) Flow cytometry analysis of infiltrating CTL in tumors. *n* = 3. d) Statistics of (c). e) Representative immunofluorescent images showing the CD3^+^CD4^+^, CD3^+^CD8^+^, and CD4^+^Foxp3^+^ cells in tumor tissues. *n* = 3. f) Statistics of (e). g) Representative RNA‐seq read coverage illustrating images of specific genes. Data are represented by mean ± SEM. Statistical significance was determined by Student's *t*‐test. ***p < 0.001, ****p < 0.0001.

SRSF1 is a splicing factor that can affect splicing and promote cancer development.^[^ ^28^, ^29^ ^]^ We evaluated SRSF1 mRNA expression in human breast cancer through TCGA database. The result revealed that SRSF1 mRNA expressed higher in cancer than that in normal tissues (Figure S6a, Supporting Information). During cancer development of the 4T1 syngeneic mouse model, expression of *Srsf1* mRNA was increased at the beginning while decreased later (Figure S6b, Supporting Information). Compared with the control 4T1 cells, 4T1 overexpressing *Srsf1* (Figure S7, Supporting Information), had stronger cell migration ability (**Figure** **6**a,b). Consistently, inoculation of 4T1 overexpressing *Srsf1* had slower tumor growth at the beginning (Figure S8, Supporting Information). In the early stage, larger number of tumor infiltrating CD3^+^CD8^+^ T cells was observed (Figure 6c,d). Meanwhile, more infiltrating T cells and less CD4^+^Foxp3^+^ Treg cells were also confirmed by tumor tissue immunofluorescent stain (Figure 6e,f). In the later stages, the immune cell infiltration was significantly compromised (Figure S9, Supporting Information). All these data suggest that *Srsf1* overexpression would promote cancer cell migration while generate neoantigen at the same time. Cancer has evolved mechanisms of both transient induction of *Srsf1* expression for metastasis and dampening the immune system simultaneously to allow these neoantigen bearing cells evading the surveillance.

We thus speculated that *Srsf1* overexpression would produce neoantigens resembling the metastatic cancer, and vaccination in advance when the immune system functioned well would prevent metastasis efficiently. As expected, *Srsf1* overexpression disturbed RNA splicing. The splicing schematic plots of representative genes showed different splicing patterns in control and *Srsf1* overexpressing cells (Figure 6g). Intron retention in typical genes analyzed by qPCR further proved that novel proteins (neoantigens) could be generated through overexpressing *Srsf1* in tumor cells (Figure S10, Supporting Information).

### 2.6 Anti‐tumor Effects of Srsf1‐Vaccine In Vivo

**Figure 7:**
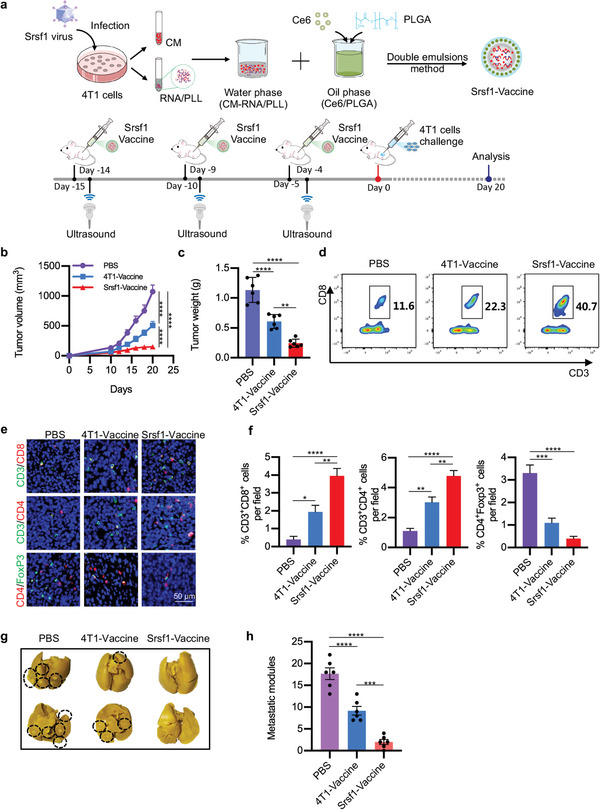
Inhibition of tumor growth and metastasis by Srsf1‐vaccine. a) Schematic illustration of Srsf1‐vaccine preparation and experimental design. b) Tumor growth cures of different groups with indicated treatments. *n* = 6. Statistical significance was determined by two‐way ANOVA with Tukey's post hoc test. c) Tumor weight in mice. *n* = 6. d) Flow cytometry analysis of infiltrating CTLs in tumors by flow cytometry. e) Representative immunofluorescent analysis of CD3^+^CD4^+^, CD3^+^CD8^+^, and CD4^+^Foxp3^+^ cells tumor tissues treated as indicated. *n* = 6. f) Statistics of (e). g) Representative images of metastasis of lungs. *n* = 6. h) Statistics of (g). Data are represented by mean ± SEM. Statistical significance was determined by one‐way ANOVA with Tukey's post hoc test. *p < 0.05, **p < 0.01, ***p < 0.001, ****p < 0.0001.

We thus prepared CM‐RNA@Ce6/PLGA using *Srsf1* overexpressed 4T1 cells. Hereafter, we named CM‐RNA@Ce6/PLGA from 4T1 cells as 4T1‐vaccine, while the particles from *Srsf1* overexpressed 4T1 cells as Srsf1‐vaccine. Then, the mice were vaccinated with 4T1‐vaccine or Srsf1‐vaccine (2 mg in 100 µL PBS), followed by 4T1 challenge (**Figure** **7**a). The tumor volume was monitored every 2 days since the 10th day after tumor‐bearing. Compared with mice immunized by PBS and 4T1‐vaccine, Srsf1‐vaccine exhibited better tumor suppression effects (Figure 7b,c). Flow cytometry results further revealed higher tumor infiltrating CTLs in Srsf1‐vaccine group (Figure 7d). Immunofluorescent staining data also confirmed that tumor tissues in Srsf1‐vaccine treated group had more infiltrating T cells and less Treg cells (Figure 7e,f). Moreover, compared with PBS and 4T1‐vaccine, Srsf1‐vaccine had better effects on preventing distant cancer metastasis (Figure 7g,h). Collectively, these results demonstrated that Srsf1‐vaccine, which contained ongoing neoantigen during cancer progression, had superiority to prevent cancer growth and metastasis than the control 4T1‐vaccine.

### 2.7 CM‐RNA@Ce6/PLGA from Cells Treated with PlaB has Similar Vaccination Effects

The above results suggested that cells with disturbed splicing pattern had superior antigens for vaccine development. To facilitate the clinical translation, we next explored if chemical compound pladienolide B (PlaB, a kind of SF3B1 regulator) had similar effects as SRSF1 in preparation of vaccine. As shown in Figure S11 (Supporting Information), qPCR analysis indicated that PlaB dose dependently interfere the splicing in 4T1 cells. Because high concentration PlaB was cytotoxic, 2.5 nm PlaB was found to be optimal for balance of cell viability and splicing disturbance (Figure S12, Supporting Information). RNA‐seq results further showed significant different usage of exon and intron after PlaB application (Figure S13a, Supporting Information). The splicing schematic plots of representative genes showed similar splicing patterns between PlaB treated cells and *Srsf1* overexpression cells (Figure S13b,c, Supporting Information).

**Figure 8:**
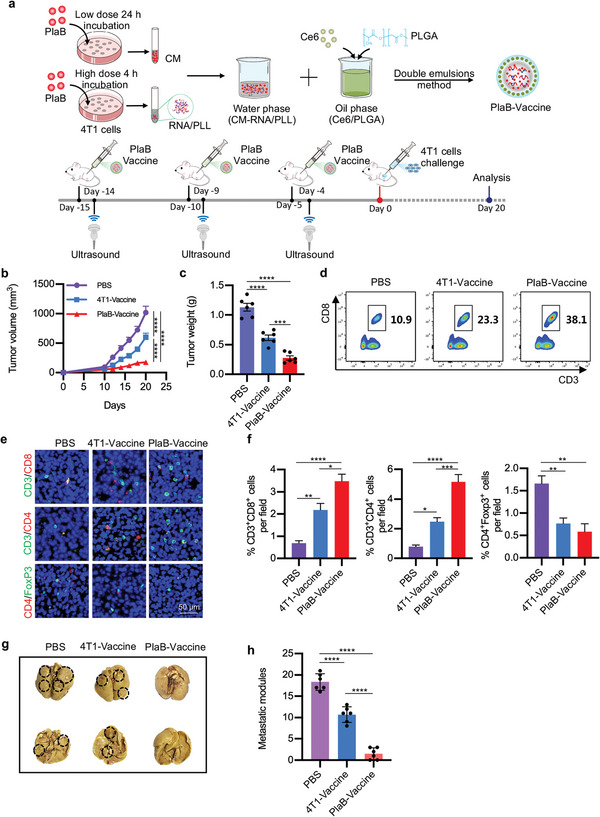
Inhibition of tumor growth and metastasis by PlaB‐vaccine. a) Schematic illustration of PlaB‐vaccine preparation and experimental design. b) Tumor growth cures of different groups with indicated treatments. *n* = 6. Statistical significance was determined by two‐way ANOVA with Tukey's post hoc test. c) Tumor weight in mice. *n* = 6. d) Flow cytometry analysis of infiltrating CTLs in tumors treated same as above. e) Representative immunofluorescent analysis of CD3^+^CD4^+^, CD3^+^CD8^+^, and CD4^+^Foxp3^+^ cells in tumor tissues treated as indicated. *n* = 6. f) Statistics of (e). g) Representative images of metastasis of lungs. *n* = 6. h) Statistics of (g). Data are represented by mean ± SEM. Statistical significance was determined by one‐way ANOVA with Tukey's post hoc test. *p < 0.05, **p < 0.01, ***p < 0.001, ****p < 0.0001.

Next, we prepared cancer vaccine using PlaB treated 4T1 cells, designated as PlaB‐vaccine, and the vaccination effects were explored in 4T1 mouse model (**Figure** **8**a). Same as before, mice were immunized with PlaB‐vaccine (2 mg in 100 µL PBS) and challenged by 4T1 cells. Compared to PBS and 4T1‐vaccine groups, PlaB‐vaccine group had better effects on tumor inhibition (Figure 8b,c). The increased number of infiltrating CTLs analyzed by flow cytometry and immunofluorescent staining also confirmed better immune activation effects in PlaB‐vaccine than that in other groups (Figure 8d–f). Moreover, PlaB‐vaccine also significantly reduced the number of metastatic nodes in lungs (Figure 8g,h). In summary, these results confirmed that PlaB‐vaccine had similar antitumor effects with Srsf1‐vaccine.

## 3 Discussion

Cancer vaccines are intensively studied in the past 10 years, emerging as a potent strategy for cancer therapy.^[^ ^30^ ^]^ Cancer vaccines typically involve selection of tumor antigens combined with adjuvants, and efficient delivery of them to DCs.^[^ ^31^ ^]^ The basic principles needed for successful therapeutic cancer vaccination include delivery of large amounts of tumor associated antigens to DCs, optimal activation of DCs, induction of effector T cell responses, and generation of immune memory.^[^ ^32^ ^]^ Currently, cancer vaccines are still challenged by the low therapeutic efficacy. The failure of cancer vaccines appears to be the low immunogenicity of the vaccine/antigens, ineffectiveness of the adjuvants, difficulty in delivery to DCs, and the suppressive tumor microenvironment preventing immune responses.^[^ ^33^, ^34^, ^35^ ^]^

Tumor antigens are essential for successful vaccine, which determine the specificity and strength of elicited adaptive immune response.^[^ ^36^ ^]^ The antigens of existing cancer vaccine strategies mainly are already known or identified from the resected cancer samples, while neoantigens of the cancer occurs during cancer progression, and thus the vaccines have rare capacity to prevent the cancer progression.^[^ ^37^, ^38^ ^]^ SRSF1 is a well characterized splicing factor that have been reported highly expressed in advanced breast cancer. In this study, the cancer cells were engineered to bear disturbed splicing by PlaB or SRSF1 overexpression, which represents an accelerated mutation (antigen generation) process during cancer evolution. Earlier exposure of these antigens confers the host advantages to control tumor growth and evolution, especially when the immune system is relatively intact and not compromised. Consistently, we here revealed that the prepared vaccine using the engineered cells had a potent capacity to prevent metastasis. Notably, the neoantigens from the engineered cells should be shared by many different cancers, as the splicing disturbance is a common characteristic of cancer.^[^ ^39^ ^]^ In other words, the proposed vaccine strategy could be also beneficial for other types of cancers. Notably, SRSF1 was studied for the proof‐of‐concept. In fact, there are also other splicing factors dysregulated expression in cancer, such as U2AF1, ZRSR2, and RBM39.^[^ ^40^ ^]^ In the future, the vaccination effects of these splicing factors are worth to be explored and compared with SRSF1.

Effective antigen presentation is very important for successful vaccination. Cancer antigens delivered by vaccination have to be taken up by DCs and presented by MHC I complex for CD8^+^ T cell priming. For vaccination, the immature DCs take up the vaccine and undergoes a maturation process. Then, they migrate to the draining lymph node and simultaneously cross‐present the protein antigens for priming of naive CD8^+^ T cells.^[^ ^14^ ^]^ The cross‐presentation is tightly regulated and disabled with tumor progression to certain extent.^[^ ^16^ ^]^ In contrast, mRNA vaccines, which naturally simulate MHC I type presentation, have superiority when applicated in cancer therapy. In this study, the vaccine proposed contain both protein and RNA antigens. Two different types of antigens used both cross‐presentation and conventional type 1 presentation mechanisms, increasing the efficiency of antigen presentation, which might explain the high efficacy of the vaccination. Antigens from lysosome degradation is essential for activation of CD4^+^ cells. In contrast, escape of the antigens, either mRNA or protein, from endosomes to the cytoplasm, is prerequisite for the subsequent presentation by MHC I, and thus activation of CTLs. In other words, endosome escape is very important in tumor immunity.

Immune adjuvants are another critical component of cancer vaccine to boost efficient anti‐tumor immunity. The current adjuvants used in cancer vaccines, such as TLR agonists, have issues of insufficient cellular immune activation and adverse effects. Different vaccine strategy needs matched adjuvant. Here, we chose Ce6, a type of sonosensitizer, as a novel cancer adjuvant candidate. In vitro and in vivo experiments both demonstrated that Ce6 together with ultrasound irradiation could booster the immune response. It has been reported that ROS is a trigger that can elicit primary immunity.^[^ ^41^, ^42^, ^43^ ^]^ One explanation might be the ROS and subsequent translation stress could promote the protein degradation by proteasome, facilitating MHC I presentation. It is reported that sonodynamic therapy could be beneficial for endosome/lysosome escape for nucleic acid delivery and subsequent gene expression, suggesting that Ce6 could be an optimal mRNA vaccine adjuvant.^[^ ^19^ ^]^ In this study, we have confirmed that CM‐RNA@Ce6/PLGA together with ultrasound irradiation could promote endosome escape and thus augment MHC I presentation in DC2.4 cells. Although DC2.4 cells have been widely used for testing the vaccination function and mechanism in vitro, confirmation of the findings in primary DCs would further provide more solid evidence.^[^ ^44^, ^45^, ^46^ ^]^

The administration time and routes also play critical roles in vaccination. With the development of cancer, the systemic immunity is impaired, as seen by the peripheral granulocytic and monocytic expansion and impaired differentiation, along with a reduction in conventional DCs.^[^ ^47^, ^48^, ^49^, ^50^ ^]^ Cancer vaccine will not work when the immune system was dysfunctional in the advanced cancer stage.^[^ ^51^ ^]^ Consistently, our study also demonstrated that with the tumor development, there were fewer infiltrating T cells in the tumor microenvironment. The decrease of CD3^+^ cells infiltration could be mainly explained by the suppressive tumor immune microenvironment, though the cancer cells might carry more neoantigens. In other words, although tumor in the advanced stage has more specific antigens, the immune response could not be elicited due to suppressive microenvironment. Hence, vaccine with these antigens at earlier stage should be beneficial. Our study here revealed that engineered cells with disturbed splicing resembles the advanced cancer and vaccination with these engineered cell derivatives could prevent metastasis effectively, tipping the balance toward eradicating cancer cells by immunity. Efficient delivery of the nanoparticles into the APCs is equally important.

As to the vaccine delivery strategy, biological vesicles are ideal carriers for cancer vaccine. Bacterial outer membrane vesicles have robust immune adjuvant effects and can elicit high level antitumor immunity.^[^ ^52^ ^]^ Meanwhile, extracellular vesicles have the advantage of easy genetic engineering, which can improve tumor microenvironment by displaying PD‐1 to block PD‐L1 on tumor cells.^[^ ^53^ ^]^ In this study, PLGA is selected as the carrier material. PLGA is a degradable polymer material approved by the Food and Drug Administration for clinical use.^[^ ^54^ ^]^ It has advantages in simultaneously loading hydrophilic and hydrophobic drugs, which is an ideal material for protein, RNA, and Ce6 loading in this study. In addition, the particle size and shape are also important factors that can affect treatment effects.^[^ ^55^ ^]^ The nanosized sphere allows the nanoparticle efficiently enriched in lymph nodes, boosting the immune activation. In addition, PLGA itself is also considered as a kind of adjuvant.

## 4 Conclusion

In summary, we here have proposed a universal method to develop cancer vaccine to prevent metastasis, which encapsulating engineered cell membrane proteins, RNAs, and sonosensitizer Ce6. The protein and RNA antigens from cells of disturbed alternative splicing resemble the antigens of metastatic cancer to certain extent, and earlier exposure of these antigens by the proposed vaccine could elicit potent immunity when the immune system is not destroyed and thus have greater capabilities to inhibit tumor growth and prevent metastasis. Assisted with ultrasound irradiation, these vaccines could stimulate DCs maturation efficiently and promote antigen presentation in multiple ways. Our work provides a novel personalized preventative cancer metastasis vaccine, which is promising for clinical translation in cancer immunotherapy.

## 5 Experimental Section

### Materials

PLGA (50:50, Mw = 7000–17 000) (719 897), PVA (Mw = 9000–10000, 80% hydrolyzed) (360 627) and poly‐l‐lysine (Mw = 15 000–30 000) (P7890) were purchased from Sigma‐Aldrich; chlorin e6 (Ce6) (21 684) was purchased from Cayman Chemical. The lipid staining kit with 1,1′‐dioctadecyl‐3,3,3′,3′‐tetramethylindotricarbocyanine iodide (DiR) (D12731) and 3,3′‐dioctadecyloxacarbocyanine perchlorate (DiO) (D275), nucleic acid stain Hoechst 33 342 (H3570), and BCA assay kit (23 227, 23 235) were purchased from Thermo Fisher Scientific. Coomassie blue fast staining solution (P0017F), SDS‐PAGE gel kit (P0012), Actin‐Tracker Green probe (C2201S), Lyso‐Tracker Green probe (C1047S), cell lysis buffer for Western and IP (P0013), and Trehalose (ST1245) were purchased from Beyotime Biotechnology Co. Ltd. First Strand cDNA synthesis Kit (4202B) was purchased from Genenode Biotech Co., Ltd. Omni‐ECL femto light chemiluminescence kit (SQ201) was purchased from Shanghai Epizyme Biomedical Technology Co., Ltd. Roswell Park Memorial Institute 1640 (RPMI 1640) culture medium, Dulbecco's modified Eagle's medium (DMEM) culture medium, fetal bovine serum (FBS) and penicillin–streptomycin solution were purchased from Cytiva Co., Ltd. CCK‐8 kits (GK10001) and DCFH‐DA (GC30006) were purchased from GlpBio technology. Luciferase Assay System (E1500) was purchased from Promega Corporation. Anti‐pan cadherin antibody (ab51034) and anti‐SF2 (ab129108) antibody were purchased from Abcam. Anti‐ATP1A1 antibody (14418‐1‐AP) and anti‐GAPDH antibody (60004‐1‐lg) were purchased from Proteintech Group, Inc. Anti‐HSP70 antibody (4872T) was purchased from Cell Signaling Technology, Inc. Collagenase I (17 100 017) was purchased from Gibco Life Technologies. Anti‐mouse H‐2Kb bound to SIINFEKL antibody‐PE (141 603), anti‐mouse CD80‐PE/Cy5 (104 712), anti‐mouse CD86‐PE (105 007), anti‐mouse CD3*ε*‐Percp (100 325), anti‐mouse CD8a‐PE (100 708), TruStain FcX (101 320), cell staining buffer (420 201), and RBC lysis buffer (420 301) for flow cytometry were purchased from BioLegend, Inc. Pladienolide B (6070) was purchased from Tocris bioscience Co., Ltd. TriPure isolation reagent (11 667 165 001) and FastStart essential DNA green master (0 692 420 4001) were purchased from Roche Co., Ltd. 4T1 cell line and HEK 293T cell line were obtained from ATCC and DC2.4 cell line was obtained from Sigma‐Aldrich.

### Animals and Cell culture

Female BALB/c mice (6–8 weeks old) were purchased from the Animal Experimental Center of the Fourth Military Medical University, and all the animal experiments were approved by the Institutional Animal Experiment Administration Committee of the Fourth Military Medical University (010 0635). All the animals were housed in specific pathogen free (SPF) conditions with 12 h/12 h light/dark cycles and provided normal commercial SPF breeding diet (Medicience, Ltd. MD17111) and autoclaved water. At appropriate times, mice were euthanized by sodium pentobarbital anesthesia followed by cervical dislocation. 4T1 and DC2.4 cells were grown in RPMI 1640 medium, and HEK 293T cells were grown in DMEM medium, containing 10% FBS and 1% penicillin–streptomycin under 5% CO_2_ humidified air at 37 °C.

### Preparation of Tumor Cell Membrane Fragments

Cell membrane was isolated as described before.^[^ ^56^ ^]^ In brief, cells were harvested by trypsin when grown to 70% confluence and washed three times using PBS, followed by centrifugation at 200 × *g* for 5 min. Then, the cells were resuspended in hypotonic lysis buffer as previous reported. Cells were disrupted using Dounce homogenizer by 40 passes under ice bath. Samples were then sequentially centrifuged at 700 × *g* for 10 min to pellet nuclei, 7000 × *g* for 30 min to pellet mitochondria, then 100 000 × *g* for 2 h to pellet membrane fragments. The membrane fragments were resuspended by ultrapure water and stored at −80 °C till use.

### Coomassie Blue Staining

Proteins in cell membrane or cells were extracted with cell lysis buffer for 30 min on ice. The protein concentrations were determined by BCA assay. Equal amounts of proteins were separated in 12% SDS‐PAGE, then stained by Coomassie Blue Fast Staining Solution.

### Western Blot Analysis

Equal amounts of protein samples were separated in 12% SDS‐PAGE, followed by transferred to nitrocellulose membrane with ice bath. The membranes were blocked with 5% bovine serum albumin for 1 h and then incubated with primary antibodies overnight at 4 °C. Antibodies used were anti‐pan cadherin, anti‐ATP1A1, anti‐GAPDH, and anti‐HSP70. After washing three times with TBS‐T (tris buffered saline‐Tween 20), and each time for 5 min, the membranes were incubated for 1 h with the corresponding secondary antibodies at room temperature and visualized using Omni‐ECL femto light chemiluminescence kit.

### Preparation of CM‐RNA@Ce6/PLGA Cancer Vaccine

The nanovaccine was prepared by double emulsion method. In brief, 100 µL RNA (6 µg µL^−1^) and 92 µL PLL (10 µg µL^−1^) were mixed and placed on ice for 30 min to form RNA particles. Then, 100 µL tumor cell membrane fragments with the protein concentration of 6 µg µL^−1^ and 8 µL ultrapure water were added into the above solution, and the mixture was considered as the water phase solution. 100 mg PLGA and 4 mg Ce6 were dissolved in 1 mL dichloromethane, forming the oil phase solution. To prepare the nanoparticles, the water phase (300 µL) was added into oil phase (1 mL), and then the mixture was sonicated by ultrasonic homogenizer (Cole Parmer 4710 Series) with 50% cycle duty for 60 s. Then, the mixture was poured into 2.5 mL PVA water solution (20 mg mL^−1^) and sonicated for another 60 s. At the end of sonification, the above mixture was added to 40 mL PVA solution (5 mg mL^−1^) and stirred for 4 h at room temperature to evaporate organic solvent. The nanoparticles were collected by centrifugation at 12 000 × *g* for 15 min and washed three times with ultrapure water. The obtained nanoparticles were resuspended using 4% trehalose, followed by lyophilization (SCIENTZ‐18N) for 48 h. For the controls, the RNA or cell membrane was replaced by water of the same volume.

### Characterization of Cancer Vaccine

The size of vaccine nanoparticles was detected by nanoparticle tracking analysis instrument (ZetaView PMX‐120) and zeta potential was detected by NanoPlus zeta/nano particle analyzer. The prepared vaccine and controls were resuspended in PBS before examination, followed by examination as instructed. The morphology of prepared cancer vaccine was analyzed by transmission electron microscope (JEM‐1400Flash).

To examine the RNA loading efficiency, the prepared nanoparticles were dissolved in DMSO, then the released RNA was extracted using RNA isolation reagent TriPure as instructed. The abundance of RNA was tested by qPCR. To evaluate the protein loading efficiency, the nanoparticles were dissolved with 0.1 m NaOH for 1 h at 90 °C, and then incubated overnight at 37 °C. Blank nanoparticles served as a negative control. After centrifugation for 15 min at 12 000 × *g*, the protein in supernatant was evaluated by BCA assay. As the nanoparticles contain PLL affect the BCA data, only these nanoparticles without PLL were tested in the study. To evaluate the Ce6 loading efficiency, the amount of Ce6 loaded into the nanoparticles was tested by UV–Vis spectrophotometer (DeNovix DS‐11) at 404 nm absorbance after the nanoparticles were dissolved by DMSO, in which Ce6 was dissolved. Encapsulation efficiency (EE) were calculated according to EE = amount of loaded materials/total amount of input ×100%.

### Cellular Uptake of Vaccine In Vitro

DCs were cultured in glass bottom confocal cell dish and incubated with CM‐RNA@Ce6/PLGA particles for indicated time. Cells were then fixed with 4% paraformaldehyde for 10 min and washed three times by PBS. To stain cytoskeleton, cells were first incubated with 0.1% Triton X‐100 in PBS and then washed four times. Then cells were stained with actin‐tracker green for 60 min under room temperature and dark condition. After washing three times with PBS, the nuclei were stained with Hoechst 33 342. Cell images were obtained by confocal laser scanning microscope (Nikon ECLIPSE Ti).

### Detection of ROS In Vitro

DCs were cultured in glass bottom confocal cell dish and incubated with 0.5 mg mL^−1^ CM‐RNA@Ce6/PLGA for 12 h. Then the cells were additionally treated with 1 W cm^−2^ ultrasound (HANIL TM HS‐501) for 1 min in groups as indicated. Then, the cells were stained with 10 µm DCFH‐DA for 30 min and washed with serum‐free medium for three times, each time for 15 s. ROS signal was detected by CLSM.

### Cell Viability and Migration Capacity

To evaluate the cell viability, cells were seeded into a 96‐well plate with 100 µL per well medium and treated with indicated. Then the cell viability was detected by CCK‐8 kit as instructed. Cell migration was evaluated by wound healing assay. Briefly, cells were seeded onto 6‐well plate, ensuring that the density reaches 100% after 24 h. A wound was made in the center of each well using a 100 µL pipette tip. Then the detached cells were removed by washing with PBS via gently shaking for three times, and each time for 15 s. After washing, cells were observed using microscope to ensure that there were no detached cells. The remained cells were then cultured with serum‐free medium. Change in wound area over time was monitored and calculated as a percentage of wound closure.

### In Vivo Distribution of the Delivered Nanoparticles

To test the in vivo distribution of the prepared nanoparticles, DiR/DiO labeled (DiR/DiO dye added into organic phase during preparation) nanoparticles (2 mg nanoparticles resuspended in 100 µL PBS) were subcutaneously injected into mice. About 12 h later, the mice were sacrificed, and heart, liver, spleen, lung, kidney, and lymph node near the injection site, were taken out for subsequent analysis. For microscopy, tissues from mice injected with DiO labeled nanoparticles were frozen for tissue sectioning. Tissues were fixed using 4% paraformaldehyde and nuclei were stained by Hoechst 33 342. Then, the fluorescent images were obtained by CLSM. For ex vivo imaging analysis, organs and lymph nodes from mice injected with DiR labeled nanoparticles were analyzed by in vivo fluorescent imaging system (Caliper IVIS Lumina II).

### Luciferase Assay

DC2.4 cells plated in 24‐well plates were incubated with 0.5 mg mL^−1^ Luci‐RNA@Ce6/PLGA. After culture for 12 h, cells were additionally treated with 1 W cm^−2^ ultrasound radiation for 1 min and further cultured for 48 h in fresh medium. After 48 h, cells were processed with luciferase cell culture lysis reagent and the supernatant protein concentration was determined by BCA assay and adjusted to 1 µg µL^−1^. Firefly luciferase activity was assayed using luciferase assay system as instructed by luminometer (GloMax 20/20).

### qPCR Analysis

Total RNA was extracted from the isolated tissues, cells, or nanoparticles with indicated treatments by TriPure reagent. Then, the RNA was reverse transcribed to cDNA using first strand cDNA synthesis kit, and the relative gene expression was analyzed using FastStart essential DNA green master by qPCR instrument (LightCycler 96). The target mRNA expression was normalized to *Gapdh*. Relative expression was calculated by normalizing to the control samples using the 2^−ΔΔ^ *^Ct^* method.

### DCs Maturation and Antigen Presentation Analysis

For DC maturation analysis, DCs were treated with different nanoparticles as indicated for 12 h. Then, cells were washed three times with PBS and cultured with fresh medium, followed by ultrasound irradiation (1 W cm^−2^) for 1 min. After additional 48 h culture, cells were blocked with TruStain FcX and stained with anti‐mouse CD86‐PE and anti‐mouse CD80‐PE/Cy5 for 20 min, followed by flow cytometry analysis (Beckman Coulter CytoFLEX).

To detect antigen presentation efficiency, cells overexpressing OVA were used to prepare CM‐RNA@Ce6/PLGA (namely OVA CM‐RNA@Ce6/PLGA). Then DC cells were incubated with control or OVA CM‐RNA@Ce6/PLGA for 12 h and treated same as above. After 48 h culture, cells were blocked with TruStain FcX and stained with anti‐mouse H‐2Kb bound to SIINFEKL antibody‐PE for 20 min, followed by flow cytometry.

### Splicing Perturbation by PlaB

4T1 cells were cultured in 10 cm cell culture dishes to 70% confluence. PlaB dissolved into DMSO, was added into culture medium. Cell RNA was isolated after 4 h culture with the PlaB final concentration of 100 nm. Cell membrane fragments were isolated after 24 h culture with the PlaB final concentration of 2.5 nm.

### Splicing Perturbation by Srsf1 Overexpression

For *Srsf1* overexpression, 4T1 cells were cultured in 24‐well plates to 20% confluence. Then cells were infected with lentiviruses in the medium containing polybrene, at MOI of 100. 16 h later, medium containing puromycin was replaced, and cells were cultured for another 72 h. The *Srsf1* overexpressed 4T1 cells were collected for other experiments.

### 5.1 Syngeneic Mouse Model

The 4T1 syngeneic mouse model was established by injecting 4T1 cells into breast pads of 6–8 weeks old female BALB/c mouse. In brief, 5 × 10^5^ 4T1 cells suspended in 100 µL PBS were injected into the fourth left mammary gland pad. To monitor the growth of tumors, the tumor volumes were measured every 2 days from day 10 after tumor inoculation. The tumor volume was measured by vernier caliper and calculated using formula: tumor volume (*V*) = 0.52 × *a* × *b* ^2^. The *a* and *b* represent the major axis (mm) and minor axis (mm) of the tumor mass.

### The Antitumor Effects of Nanovaccines

The mice were vaccinated by subcutaneous injected by nanoparticles (2 mg nanoparticles resuspended in 100 µL PBS) for three times as indicated. 20 days after 4T1 challenged, tumors were harvested and cut into small pieces, followed by digestion with 200 units mL^−1^ collagenase I (in HBSS buffer) for 40 min. Cell suspensions were filtered by 200‐mesh gauze, and red blood cells were then lysed by RBC lysis buffer. The isolated cells were blocked by TruStain FcX and stained with anti‐mouse CD3*ε*‐Percp and anti‐mouse CD8*α*‐PE before flow cytometry.

### Statistical Analysis

All the data are expressed as mean ± SEM. Student's *t*‐test was used for comparison of two groups while one‐way ANOVA with Tukey's post hoc test for multiple groups. The statistical differences of tumor growth over time were calculated by two‐way ANOVA with Tukey's post hoc test. All statistical analyses were done using GraphPad Prism 7.0. Significant differences are considered at *p* < 0.05.

## Conflict of Interest

The authors declare no conflict of interest.

## Supporting information

Supporting Information

## Data Availability

The data that support the findings of this study are available from the corresponding author upon reasonable request.
