# Supplementary material for: Ultrasound Responsive Nanovaccine Armed with Engineered Cancer Cell Membrane and RNA to Prevent Foreseeable Metastasis
Source: Adv Sci (Weinh). 2023 Apr 25;10(19):2301107. doi: 10.1002/advs.202301107 (PMC10323640; doi:10.1002/advs.202301107)
Supplement: Supplementary file 1 — Supporting Information [file ADVS-10-2301107-s001.pdf]

ADVANCED SCIENCE
Open Access

Supporting Information

for *Adv. Sci.*, DOI 10.1002/advs.202301107

Ultrasound Responsive Nanovaccine Armed with Engineered Cancer Cell Membrane and RNA to Prevent Foreseeable Metastasis

Wenqi Sun, Panpan Ji, Tian Zhou, Zhelong Li, Changyang Xing, Liang Zhang, Mengying Wei, Guodong Yang and Lijun Yuan**

Supporting Information

Ultrasound responsive nanovaccine armed with engineered cancer cell membrane and RNA to prevent foreseeable metastasis

Wenqi Sun, Panpan Ji, Tian Zhou, Zhelong Li, Changyang Xing, Liang Zhang, Mengying Wei, Guodong Yang, and Lijun Yuan**

W. Sun, Z. Li, T. Zhou, C. Xing, L. Zhang, L. Yuan
Department of Ultrasound Diagnostics
Tangdu Hospital, Fourth Military Medical University, Shaanxi, 710038, China
E-mail: yuanlj@fmmu.edu.cn
P.Ji
Department of Digestive Surgery
Xijing Hospital, Fourth Military Medical University, Shaanxi, 710032, China
W. Sun, Z. Li, M. Wei, G. Yang
The State Laboratory of Cancer Biology
Department of Biochemistry and Molecular Biology, Fourth Military Medical University, Shaanxi, 710032, China
E-mail: yanggd@fmmu.edu.cn

Keywords: cancer vaccine, alternative splicing, antigen presentation, sonosensitizer

Table S1 Sequences of qPCR primers used in the study

Gene name	Forward primer (5'-3')	Reverse primer (5'-3')
Psmb8	CATTCCTGAGGTCCTTTGGTGGTG	ACTTGAAGGCGAGTGTGGTTGTG
Tap1	GGACTTGCCTTGTTCCGAGAG	GCTGCCACATAACTGATAGCGA
Srsf1	GCGGTCCGAGAACAGAGT	CGGTAAACATCAGCGTAACATA
Nudt14	TCTTCCCGTCTGAGCAA	CACGATCAAGGGCCTAA
Dnajc11	TGGCTTCCCGCAGATT	CCTTCCCTCAGTGGCTAA
Dnajb1	TGTGAGAATAATGGGTTGTGG	GCAGTGGCTGGCTTAGGA
Cdkn1b	CTCGTCAGACAATCCGGCT	CGGAGACAGACAGACAGACA
Gapdh	AGGTCGGTGTGAACGGATTTG	TGTAGACCATGTAGTTGAGGTCA

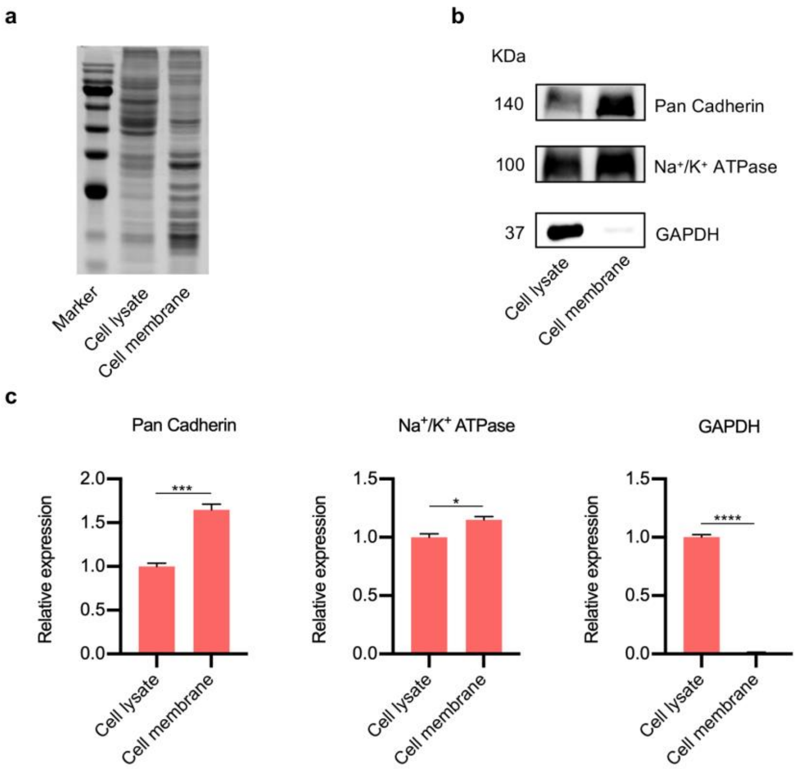

Figure S1 Characterization of cell membrane. (a) SDS-PAGE analysis of the protein components of the cell membrane isolate. Gel was stained by Coomassie blue and cell lysates served as control. (b)Western blot analysis of cell membrane markers. (c) Statistics of western blot data. Data are shown as mean ± SEM representative of 3 different experiments. Statistical significance was determined by Student's *t*-test. **p* < 0.05, ****p* < 0.001, *****p* < 0.0001.

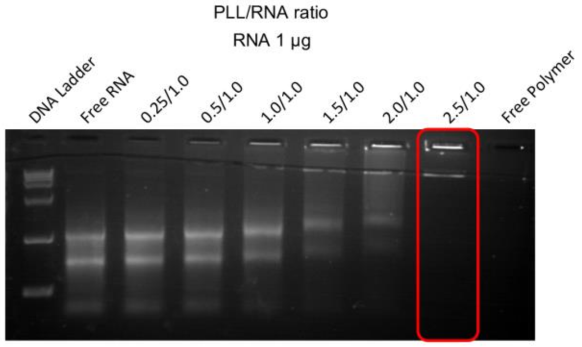

Figure S2 The UV fluorescent image of RNA gel retardation assay. RNA was concentrated with poly-lysine in different PLL/RNA ratios.

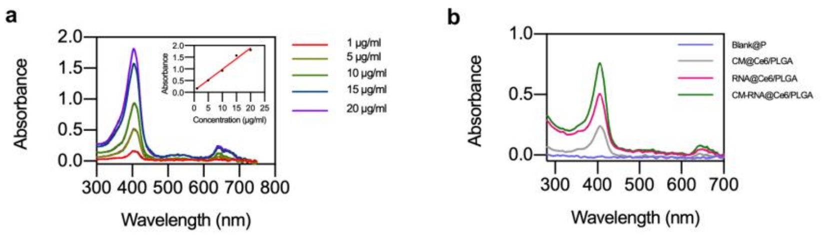

Figure S3 Quantification of encapsulated Ce6 by UV-Vis. (a) The absorbance of Ce6 detected by UV-Vis spectra and the standard curve. (b) The absorbance of the Ce6 encapsulated in the indicated nanoparticles detected by UV-Vis spectra.

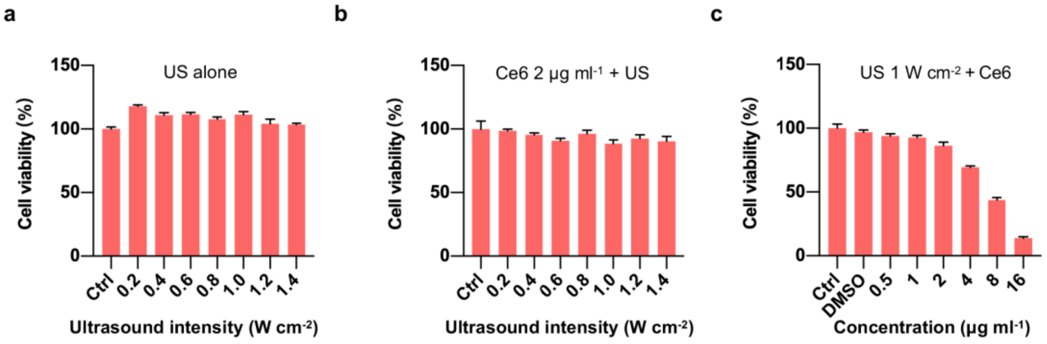

Figure S4 Cell toxicity in groups treated with different doses of ultrasound and Ce6. (a) CCK-8 assay showing the cell viability of DCs treated by ultrasound with different intensity. (b) CCK-8 assay evaluating cell viability of DCs treated with 2 μg ml^{-1} Ce6 plus ultrasound with different intensities. (c) CCK-8 assay evaluating cell viability of DCs treated with 1 W cm^{-2} ultrasound plus different doses of Ce6. Data are represented by mean ± SEM, n = 6.

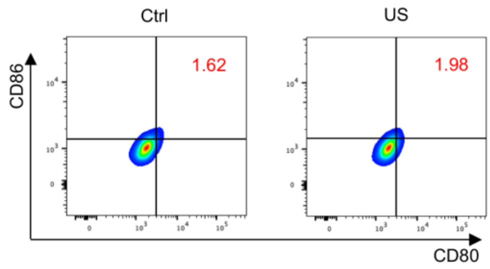

Figure S5 Flow cytometry analysis of DC activation in cells treated with or without ultrasound irradiation. DCs were untreated or subjected to ultrasound irradiation, and the maturation analyzed by flow cytometry.

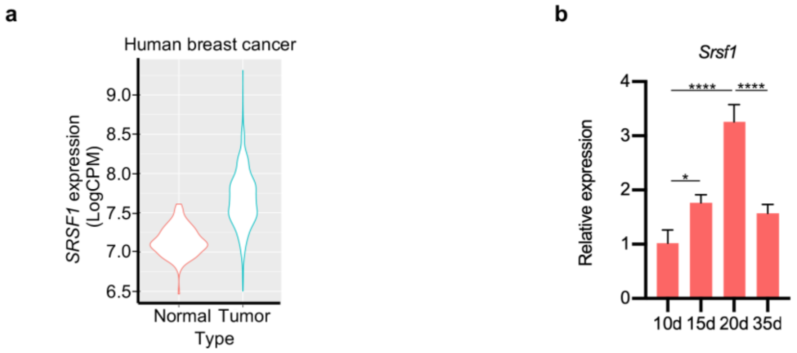

Figure S6 *Srsf1* expression in breast cancer. (a) *Srsf1* gene expression in human breast cancer analyzed by TCGA database. (b) Relative *Srsf1* gene expression in 4T1 tumor tissues tested by qPCR. n = 3. Data are represented by mean ± SEM. Statistical significance was determined by one-way ANOVA with Tukey's post hoc test. *$p < 0.05$, ****$p < 0.0001$.

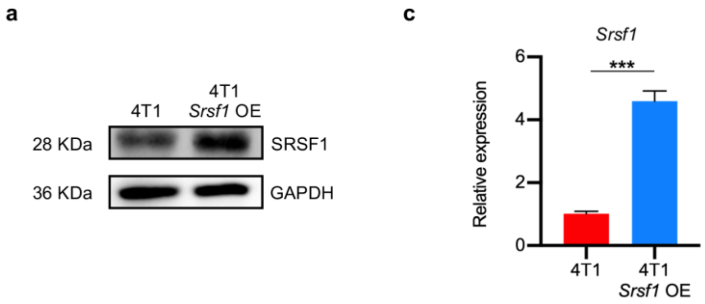

Figure S7 SRSF1 overexpression efficiency confirmed by western blot and qPCR. (a) Western blot analysis of SRSF1 protein expression in 4T1 cells and 4T1 *Srsf1* OE cells. (b) qPCR analysis of *Srsf1* mRNA expression in cells. n = 3. Statistical significance was determined by Student's *t*-test. Data are represented by mean ± SEM, ***$p < 0.001$.

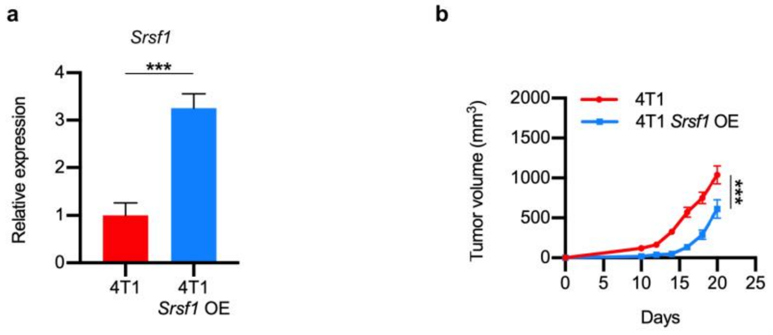

Figure S8 *Srsf1* expression dynamically regulates tumor growth. (a) qPCR analysis of *Srsf1* mRNA expression in tumor tissues. n = 6. Statistical significance was determined by Student's *t*-test. (b) Tumor volumes at different times after inoculation. n = 6. Statistical significance was determined by two-way ANOVA. Data are represented by mean ± SEM. ***$p < 0.001$.

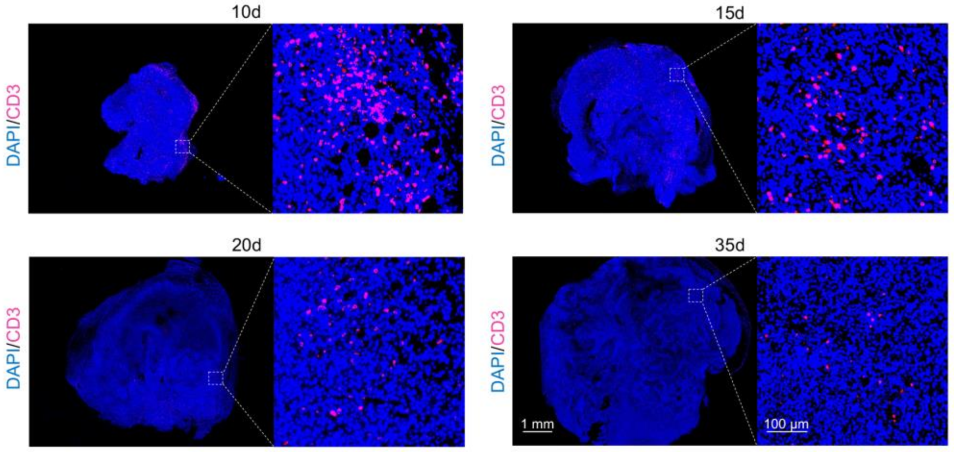

Figure S9 Immunofluorescent images showing the $CD3^+$ cells in 4T1 tumor tissues during tumor progression. Scale bar = 100 μm

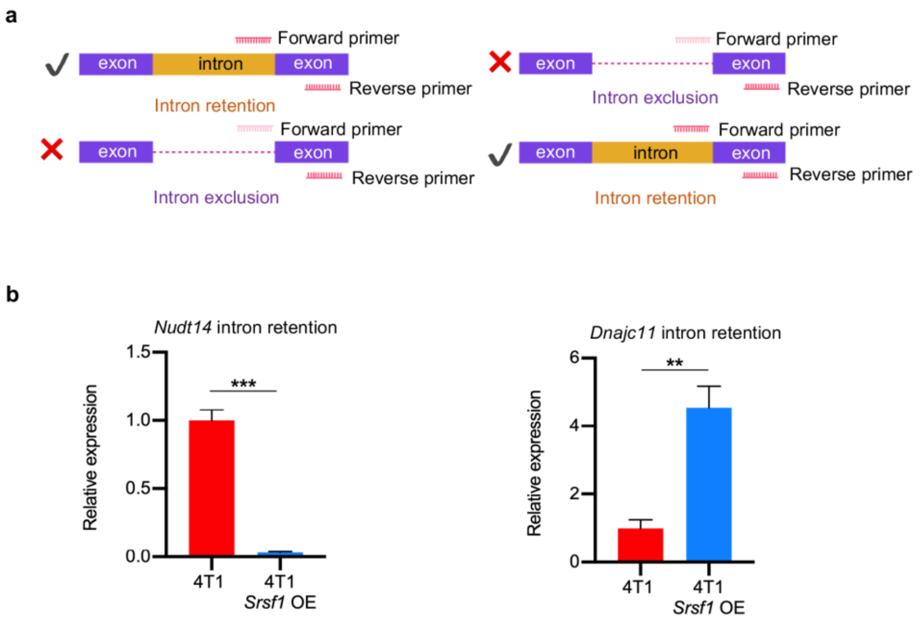

Figure S10 *Srsf1* overexpression alters splicing. (a) Schematic of alternative splicing analyzed by qPCR. (b) qPCR analysis of altered splicing of interested genes in control and 4T1 *Srsf1* OE cells. n = 3. Data are represented by mean ± SEM. Statistical significance was determined by Student's *t*-test. $^{**}p < 0.01$, $^{***}p < 0.001$.

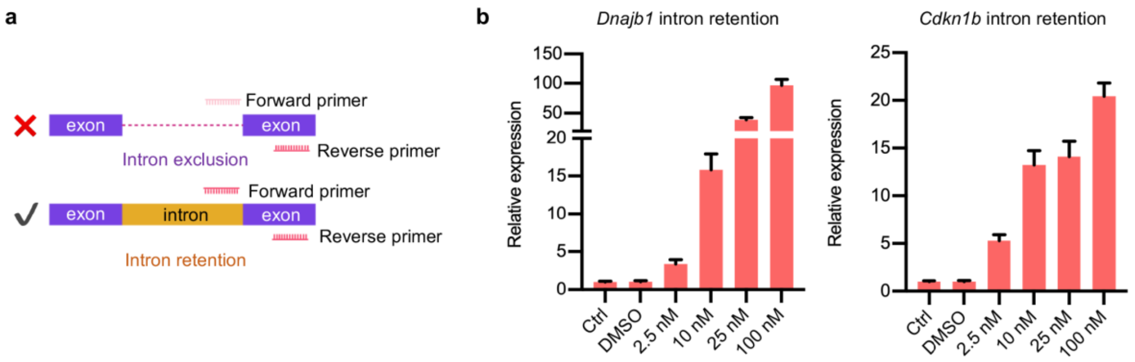

Figure S11 PlaB treatment alters splicing. (a) Schematic of alternative splicing analyzed by qPCR. (b) qPCR analysis of altered splicing of interested genes in control and PlaB treated 4T1 cells. Data are represented by mean ± SEM, n = 3.

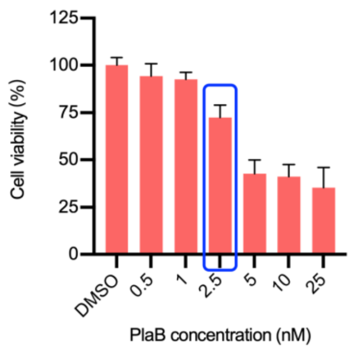

Figure S12 CCK-8 assay evaluating cell viability in 4T1 cells treated with PlaB at different doses. Data are represented by mean ± SEM. n = 3.

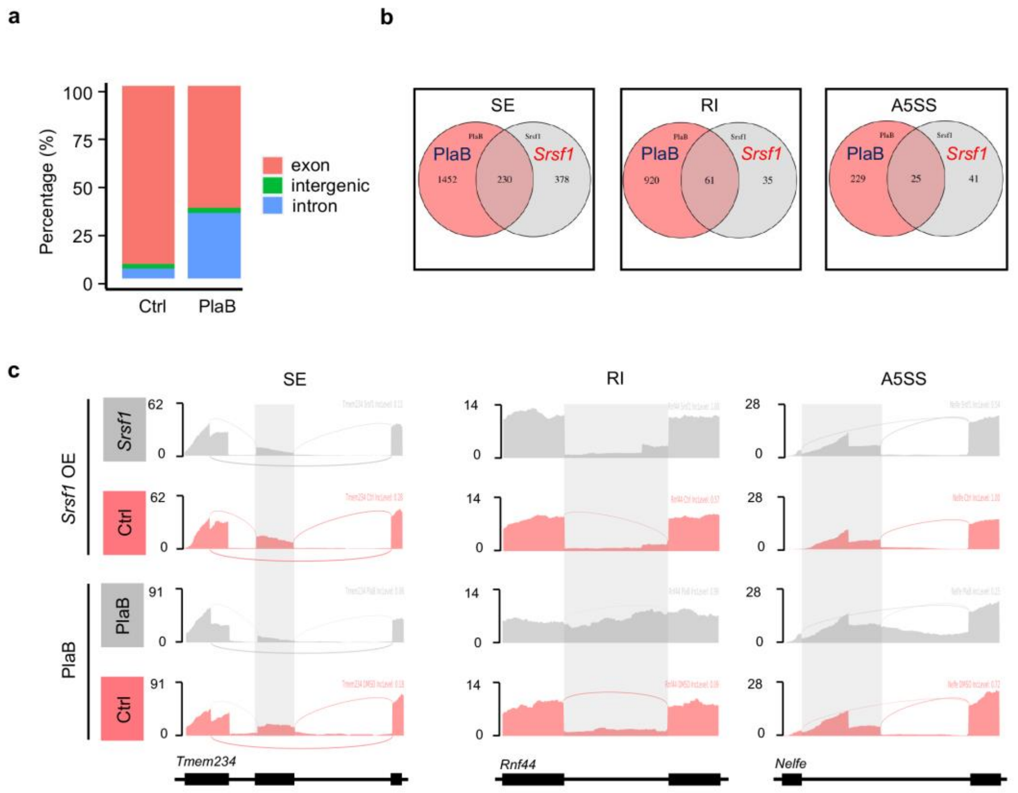

Figure S13 Comparison of altered splicing in PlaB treated cells and *Srsf1* overexpressing cells. (a) Ratio of exon, intron, and intergenic region in Ctrl and PlaB treated 4T1 cells. (b) Veen diagrams of gene number with SE, RI, and A5SS splicing pattern in PlaB treated cells and Srsf1 overexpressing cells. (c) Representative RNA-seq read coverage illustrating images of specific genes. SE: skipping exon, RI: retained intron, A5SS: alternative 5’ splice site.
